# Biosorption Potential of *Bacillus salmalaya* Strain 139SI for Removal of Cr(VI) from Aqueous Solution

**DOI:** 10.3390/ijerph121214985

**Published:** 2015-12-03

**Authors:** Arezoo Dadrasnia, Kelvin Swee Chuan Wei, Nasser Shahsavari, Mohd Sofian Azirun, Salmah Ismail

**Affiliations:** 1 Department of Biohealth Science, Institute of Biological Sciences, Faculty of Science, University of Malaya, 50603 Kuala Lumpur, Malaysia; are.dadrasnia@gmail.com (A.D.); scwkelvin@hotmail.com (K.S.C.W.); 2 Department of Biological Science, College of Agricultural and Environmental Engineering, Islamic Azad University, Hajiabad Branch, Hormozgan 55773, Iran; shahsawari110@gmail.com; 3 Institute of Biological Sciences, Faculty of Science, University Malaya, 50603 Kuala Lumpur, Malaysia; sofian@um.edu.my

**Keywords:** *Bacillus salmalaya*, bioremediation, chromium, kinetic, isotherm

## Abstract

The present study investigated the biosorption capacity of live and dead cells of a novel *Bacillus* strain for chromium. The optimum biosorption condition was evaluated in various analytical parameters, including initial concentration of chromium, pH, and contact time. The Langmuir isotherm model showed an enhanced fit to the equilibrium data. Live and dead biomasses followed the monolayer biosorption of the active surface sites. The maximum biosorption capacity was 20.35 mg/g at 25 °C, with pH 3 and contact time of 50 min. Strain 139SI was an excellent host to the hexavalent chromium. The biosorption kinetics of chromium in the dead and live cells of *Bacillus salmalaya* (*B. salmalaya*) 139SI followed the pseudo second-order mechanism. Scanning electron microscopy and fourier transform infrared indicated significant influence of the dead cells on the biosorption of chromium based on cell morphological changes. Approximately 92% and 70% desorption efficiencies were achieved using dead and live cells, respectively. These findings demonstrated the high sorption capacity of dead biomasses of *B. salmalaya* 139SI in the biosorption process. Thermodynamic evaluation (ΔG^0^, ΔH^0^, and ΔS^0^) indicated that the mechanism of Cr(VI) adsorption is endothermic; that is, chemisorption. Results indicated that chromium accumulation occurred in the cell wall of *B. salmalaya* 139SI rather than intracellular accumulation.

## 1. Introduction

**Scheme 1:**
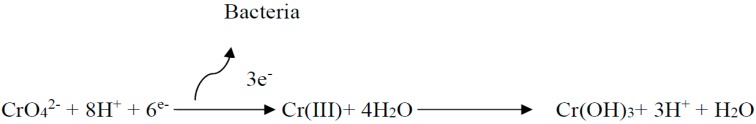
Process of chromium reduction during the biological treatment.

The bioaccumulation tendency and toxicity of the present heavy metals, such as zinc, mercury, lead, chromium, and cadmium, pose a major threat to the environment [1]. Chromium is one of the most important hazardous heavy metals in wastewater; this element does not easily degrade and is highly resistant to oxidation even at high temperatures [2]. Chromium is widely distributed in the environment, particularly among textile industries, petroleum refining, and chemical and electronic manufacturing, as well as agriculture and mining activitie. Chromium is released into the environment through leaching of toxic ingredients, improper disposal practices, leakage, and poor storage tanks, resulting in contaminated ground water and soil at production sites. Chromium pollution poses a serious threat to fish, aquatic biodiversity, and human health. In accordance with the drinking water guidelines, the maximum recommended limit for total Cr is 0.05 mg/L [3]. Excessive amount of chromium causes headache, nausea, vomiting, as well as skin, throat, and lung cancers [3]. In nature, chromium is present as Cr(VI) or Cr(III), which differ in biochemical reactions and physicochemical properties [4]. For instance, Cr(VI) is found as chromate (CrO_4_^2−^) and considered as soluble, mobile, toxic, and carcinogenic with a potential risk for surface water contamination [5]. By contrast, Cr(III) is immobile, less toxic, and readily precipitating as Cr(OH)_3_. Cr(III) is an essential element in the metabolism of lipids and carbohydrates [6]. Various conventional chemical and physical methods have been applied to remove heavy metals from the environment; these methods include ion exchange, photo catalytic reduction, precipitation, ultrafiltration, and reverse osmosis [7,8,9]. These processes lead to incomplete metal removal, which is influenced by several criteria, and the production of secondary contamination, high-energy consumption, and high operating cost [2,10]. Thus, cost-effective biological techniques that are efficient in removing heavy metals should be developed. In this regard, algae, yeast, bacteria, and fungi have been proven capable of binding and adsorbing metal ions, as well as helping in catalyzing chemical reactions [11,12,13]. Recently, the focus has been drawn on the application of bacterial biomass as a biomaterial for removing metals from wastewater. Based on the metabolic activity and presence of different functional groups and proteins on bacterial cell walls, such as sulfate, amine, hydroxyl, and phosphates, the microbes interact with metals and can accumulate heavy metal ions on their surface area [14]. When the microorganisms reduce Cr(VI), a large amount of protons can reduce Cr(VI) into trivalent form [Cr(III)]. The reduction process during the biological treatment is described in Scheme 1 [15].

However, various studies have reported the use of fungi, yeast, and bacteria either as live or dead cells for the removal of heavy metals [15]. Benazir *et al.* [16] reported the ability of a consortia of indigenous bacteria to reduce Cr(VI) at various concentrations from 100 mg/L to 300 mg/L. *Bacillus subtilis*, *Pseudomonas aeruginosa*, and *Saccharomyces cerevisiae* in consortia and in their immobilized forms reduced 770 mg/L of Cr(VI) before remediation to 5.2 mg/L to 5.7 mg/L [17]. Many Cr pure and enriched mixed cultures of microorganisms are illustrated to detoxify Cr(VI) during bioreduction (Cr(VI) to Cr(III)) under aerobic and anaerobic conditions [18]. Ye *et al.* [19] demonstrated that red algae *Porphyra leucosticte* as a biodegradable sorbent has maximum bioremediation capacity of metal ions. They reported that *P*. *leucosticte* biodegraded 31.45 mg/g of Cd(II) and 36.63 mg/g of Pb(II) at biomass dosage 15 g/L, pH 8.0 and contact time 120 min containing initial 10.0 mg/L of Cd(II) and 10.0 mg/L of Pb(II) solution. The resulting bioremediated water from heavy metal using *Pseudomonas aeruginosa* has been tested with a live albino rat for forty days [20].

*Bacillus salmalaya* 139SI has shown a high potential of remediating hydrocarbons from contaminated soil and water [21,22,23]. However, the current study aimed to evaluate the Cr(VI) biosorption potential from the dead and live cells of a novel *Bacillus* strain isolated from an agricultural soil in Malaysia. This biosorption batch study was conducted to assess the maximum rate of biosorption of Cr(VI), and the optimum conditions were determined in various pH, initial Cr(VI) concentration, and time. A kinetic model and some adsorption isotherms were applied. Thermodynamic parameters were also calculated. At the end of the experiments, dead and live biomass cells were analyzed by scanning electron microscopy (SEM), energy dispersive X-ray spectrometry (EDX), and Fourier transform infrared (FTIR) to identify the cells with significant advantages in Cr(VI) biosorption.

## 2. Materials and Methods

### 2.1. Microorganism and Growth Conditions 

Strain Salmah Ismail (SI) 139SI was originally isolated from the soil obtained from a private farm in Selangor, Malaysia (2.99917° N 101.70778° E) [24]. The obtained soil sample was suspended in sterile distilled water and streaked on the Brain–Heart Infusion (BHI) agar plates supplemented with 5% sheep blood and incubated for 24 h at 37 °C. The 139SI strain was an isolate that exhibited strong hemolytic activity. Thus, this strain was routinely cultured on BHI blood agar and maintained in a glycerol suspension (25%, w/v) at −80 °C and on BHI-slant agar at room temperature.

### 2.2. Identification of Bacteria Using 16S rRNA 

The 16S rRNA molecular DNA sequencing technique was performed on the isolate. For genomic DNA extraction, the bacterial isolate was cultured in 10 mL of BHI broth overnight at 150 rpm in a 37 °C shaking incubator. Bacterial cells were harvested, and their genomic DNA was extracted using NucleoSpin Tissue in accordance with the manufacturers’ instructions. The presence of genomic DNA was further confirmed by 0.7% agarose gel electrophoresis. The selected 16S rRNA universal primers 27F (5’-AGAGTTTGATCMTGGCTCAG-3’) and 1492R (5’-GGTTACCTTGTTACGACTT-3’) were used to amplify the 16S rRNA region. The complete 16S rRNA DNA sequence was built through contig assembly by using VectorNTi and compared with other DNA sequences in the GenBank database through the BLASTN program.

### 2.3. Minimum Inhibitory Concentrations and Antibiotic Resistance of Strain 139SI 

The minimum inhibitory concentration (MIC) is the lowest concentration that prevented the bacterial growth; in this study, MIC was determined through turbidimetric analysis [25,26]. The stock solution of CrCl_3_ was prepared in double-distilled water and added to the nutrient broth in various concentrations (30 ppm to 150 ppm). Each tube was inoculated with approximately 1.5 × 10^6^ colony forming units/mL cells. The optical density (600 nm) was measured after 24 h incubation at 37 °C. The MIC values of Ciprofloxacin, Amikacin, Doripenem, Ampicilin, Linezolid, and Aztreonam were determined on Trypticase soy agar plates by using MIC trat strip antibiotic sensitivity test. The zone of inhibition was noted after 24 h incubation. The result was compared with that of the European Committee on Antimicrobial Susceptibility Testing (EUCAST) database.

### 2.4. Preparation of Live and Dead Cells 

The *B*. *salmalaya* strain 139SI was inoculated into BHI medium at 37 °C and 150 rpm agitation in a shake flask cultured for 72 h. The cells were grown to the late exponential phase, and live cells were harvested by centrifugation at 8000 rpm for 20 min at 4 °C, whereas the dead cells were harvested after autoclaving at 121 °C for 20 min [27]. Pellets were washed with deionized water, and the cells were prepared as biosorbents for Cr(VI) biosorption.

### 2.5. Batch Biosorption Experiments

The effect of time, pH (2.0 to 8.0), and concentration of chromium during the biosorption process were examined. Live and dead pellets of 1 g/L were mixed with an initial Cr(VI) concentration of 50 ppm (prepared from potassium dichromate) at 30 ± 2 °C and agitated at a speed of 150 rpm. Biosorption studies were also conducted with different concentrations of chromium (20, 40, 60, 80, 100, 120, 140 and 160 mg/L) at pH 3.0. Samples were obtained at definite intervals (5, 10, 20, 30, 40, 50, 75, 100, 125, 150, 200, 250, 300, and 350 min) for the residual metal ion concentrations in the solution. The final concentration of chromium in the supernatant was determined by inductively coupled plasma optical emission spectrometry (ICP-OES) at 8000 rpm for 20 min. The values of biosorptive capacity of chromium was calculated as follows (Equation (1)):

Q_e_ = (C_α_−C_β_)/C_biomass_ (1)

where Q_e_ is the metal uptake (mg metal per g biosorbent), C_biomass_ is the biomass concentration (g cell/L), C_α_ is the initial metal concentration, and C_β_ is the equilibrium metal concentration (mg/L). The biosorption experiments were conducted in triplicate.

### 2.6. Desorption Efficiency

The desorption experiment was carried out to find the efficiency and reusability of biosorbent. After the biosorption experiments, the adsorbed chromium on to the live and dead biosorbents were separated from the solution by filter paper (Whatmann no. 1). Then, the separated biosorbent was added to metal-free solutions (0.1 N HCl, HNO_3_ and 0.1 M EDTA) and kept one hour before determining the final chromium concentration. After each cycle, the biosorbent was washed with ddH_2_O water, before successive cycles. Each new cycle of adsorption was carried out by supplementing of 100 mg/L chromium with different pH (Equation (2)).

DE (%) = Mr/Ma × 100
(2)

where M_r_ represents the amount of chromium released into the supernatant (mg), and M_a_ is the initial amount of adsorbed Cr (mg).

### 2.7. Biosorption Equilibrium

To calculate the adsorption isotherm, two equilibrium models were applied, namely, Langmuir and Freundlich. These models described the nature of the adsorption process and the adsorption capacity of chromium onto live and dead biomasses of *B. salmalaya* 139SI, which were expressed in terms of the quantity of metal adsorbed per unit of mass of adsorbent used (mmol/g or mg/g) [25]. The Langmuir model predicted a monolayer adsorption using a kinetic approach that is conducted as a single layer placed onto surface cells and described as follows [25,28] (Equation (3)):

Q_e_ = Q _max_ (βC_e_ /1) + β C_e_ or C_e_/ Q_e_ = 1/βQ _max_ + (1/ Q_max_) C_e_ (3)

Q_e_ = amount adsorbed metal per unit weight biomass at equilibrium (mg/g)
Q_max_ = maximum adsorption capacity (mg/g)
β = equilibrium constant for adsorption (L/mg)
C_e_ = residual metal concentration in solution (mg/l)

In a heterogeneous biosorption system, Freundlich isotherm was used for modeling as follows (Equation (4)):

Q_e_ = K_f_ C_e_^1/n^ or logQ_e_ = log K_f_ +1/n log C_e_ (4)

where K_f_ (L/g) is the isotherm constants of the Freundlich equation, and *n* indicates the intensity of the sorbent and the effect of the metal ion concentrations. The linearization of both models was integrated into the experimental isotherm data. Table 2 summarizes the adsorption constants (Q_max_, β, K_f_, and *n*) from the Langmuir and Freundlich isotherms with correlation coefficients (*R*^2^). The biosorption process is defined in terms of the value of the two constants, β and Q_max_ [13,29]. β represents the initial sorption isotherm slope, whereas Q_max_ cannot be reached at experimental conditions. However, the high β and Q_max_ are favorable for a good biosorbent. 

### 2.8. Biosorption Thermodynamics

To determine the influences of heat change (20 °C, 30 °C, 40 °C, 50 °C, 60 °C, and 80 °C) in the biosorption process, thermodynamic parameters were calculated as follows (Equation (5)):

∆G^0^ = ∆H^0^−T ∆S^0^,
∆G^0^ = −RT lnK,
ln K = (∆S^0^/R)−(∆H^0^/RT)
K = Q_e_/ C_e_ (5)

where ΔG^0^, ΔH^0^ , and ΔS^0^ are the Gibbs free energy, enthalpy, and entropy, respectively. R is the gas constant (8.314  J/mol·K), K is the distribution coefficient, and T is the temperature (K).

### 2.9. Kinetic Studies

A quantitative understanding of the sorption is possible with the help of kinetic models. Kinetic study predicts the efficiency of the biosorbent and the mechanisms of biosorption. Kinetic study also explains the characteristics of the adsorption process for its successful application [30]. When considering a proper model, two different kinetic models, namely, first-pseudo (6) (Equation (6)) and second-pseudo (7) (Equation (7)) orders, were investigated.

log (Q_e_ − Q_t_) = logQ_e_ – (K_1_/2.303) t (6)

1/Q_t_ = 1/K_2_ Q_e_^2^ + (1/Q_e_) t (7)

K_1_ (L/min) and K_2_ (g/mg min) = rate constants of the respective kinetic models
Q = sorption capacities at equilibrium (mg/g)
Q_e_ = sorption capacities at time t (mg/g)

### 2.10. FT-IR, EDS, and SEM Analysis

Surface characterization of the adsorbent was determined using FT-IR. Dead and live biosorbents that were loaded with chromium were analyzed using spectrum 4000, US. Free- and metal-treated absorbents were dried and mixed with potassium bromide powder at a ratio of 1:100 and pressed under 10 tons of pressure. The prepared pellets were measured between 600 and 4000 cm^−1^. The EDS and SEM of the adsorbent before and after the adsorption of chromium were recorded using FESEM (SEI quanta SEG 450 model, Netherlands).

### 2.11. Bioaccumulation of Chromium in *B. salmalaya* 139SI

The following method was conducted to determine the chromium bioadsorption on the cell surface and the chromium accumulation inside the cell. Approximately 0.1 g dry cells were cultured (at 30 °C for 24 h with 150 rpm agitation) in nutrient broth that contained different concentrations of chromium. Samples were harvested after centrifugation at 8000 rpm for 15 min. The pellets were washed with double-distilled water and suspended into 0.01 M sterilized EDTA for 15 min to remove metal ions bound to cell wall components. After further centrifugation (at 8000 rpm for 15 min), the supernatant was analyzed to determinate the extent of cell wall-bound chromium ions. In order to determine the bioaccumulated chromium ions inside the cell, pellets were analyzed after HNO_3_ digestion in a microwave oven. 

### 2.12. Statistical Analyses

To evaluate the statistical results, a general linear model (SPSS 19) was used for the ANOVA between the means of the treatments. In addition, Duncan’s multiple range test was performed to test of significance (*p* < 0.05).

## 3. Results and Discussion

### 3.1. Molecular Identification of Bacterial Isolate Using 16S rRNA

**Figure 1:**
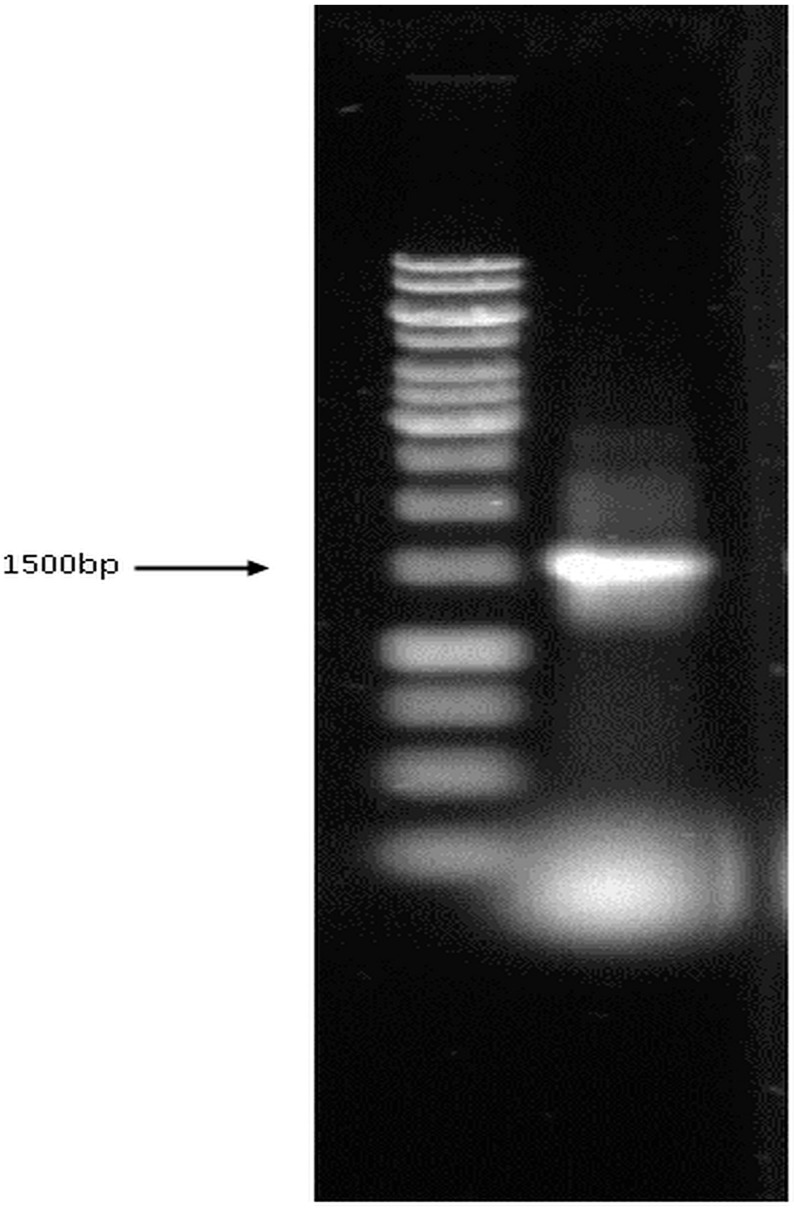
PCR amplification of 16S rRNA gene.

**Figure 2:**
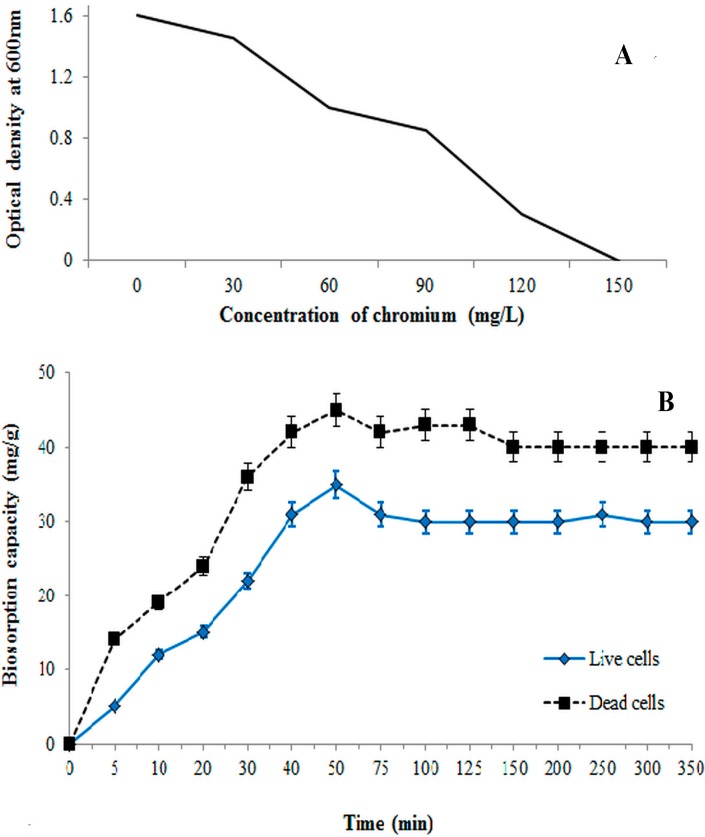
(**A**) Minimum inhibitory concentrations of *B. salmalaya* 139SI; (**B**) Effect of contact time. Vertical bars indicate SE (*n* = 3).

The bacterial isolate was confirmed using 16S rRNA sequencing based on the comparison of the highly conserved 16S rRNA gene. The amplified DNA sequence was approximately 1500 bp, which is based on the observation of a visible single band in the agarose gel (Figure 1). The band was excised and sent for DNA sequencing through Genomics BioScience and Technology (Taiwan) with the 27F and 1492R(l) universal primers using Applied Biosystems 3730xl DNA Analyzer. The 16S rRNA sequence was assembled and searched using BLAST with the NCBI GenBank, and the results indicated that the bacterium belongs to the *Bacillus* genera (Table 1). Bacterial growth stopped at 150 ppm; thus, this concentration was considered as the MIC (Figure 2A). The result of antibiotic resistance shows that the bacterium was most resistant to Ciprofloxacin, Amikacin, Doripenem, Ampicilin, and Linezolid. However, this bacterium was susceptible to Aztreonam, as compared with the reports in the EUCAST database.

**Table 1:** BLAST results of bacterial isolate.

Description	Max Score	Total Score	Query Cover	E Value	Ident	Accession
*Bacillus salmalaya* strain 139SI 16S ribosomal RNA gene, partial sequence	2719	2719	100%	0.0	100%	KM051837.1
Uncultured bacterium clone 11 St 10 16S ribosomal RNA gene, partial sequence	2713	2713	100%	0.0	99%	KM464089.1
Uncultured bacterium clone EGSB 200 5-5 16S ribosomal RNA gene, partial sequence	2713	2713	100%	0.0	99%	KJ881337.1
*Bacillus anthracis* str A16, complete genome	2713	29732	100%	0.0	99%	CP001970.1
*Bacillus* sp. C20 16S ribosomal RNA gene, partial sequence	2713	2713	100%	0.0	99%	KF479614.1
*Bacillus* sp. B10 16S ribosomal RNA gene, partial sequence	2713	2713	100%	0.0	99%	KF479574.1
*Bacillus* sp. A52 16S ribosomal RNA gene, partial sequence	2713	2713	100%	0.0	99%	KF479557.1
*Bacillus cereus* strain XX2010 16S ribosomal RNA gene, partial sequence	2713	2713	100%	0.0	99%	JX993816.1
*Bacillus cereus* F837/76, complete genome	2713	32440	100%	0.0	99%	CP003187.1
*Bacillus cereus* strain 7D, 16S ribosomal RNA gene, partial sequence	2713	2713	100%	0.0	99%	JF714217.1
*Bacillus* sp, LM24(2011)16S ribosomal RNA gene, partial sequence	2713	2713	100%	0.0	99%	HQ891939.1
*Bacillus thuringiensis serovar finitimus* YBT-020, complete genome	2713	37883	100%	0.0	99%	CP002508.1
*Bacillus cereus* strain PPB13 16S ribosomal RNA gene, partial sequence	2713	2713	100%	0.0	99%	HM771668.1
*Bacillus cereus* strain SBD1-8 16S ribosomal RNA gene, partial sequence	2713	2713	100%	0.0	99%	HQ236038.1
*Bacillus cereus* strain DZ-h 16S ribosomal RNA gene, partial sequence	2713	2713	100%	0.0	99%	HM345997.1
*Bacillus thuringiensis* strain, IWF24 16S ribosomal RNA gene, partial sequence	2713	2713	100%	0.0	99%	GU120652.1
*Bacillus cereus* 03BB102, complete genome	2713	37938	100%	0.0	99%	CP001407.1
*Bacillus cereus* AH820, complete genome	2713	32444	100%	0.0	99%	CP001283.1
*Bacillus* sp. NS-4 16S ribosomal RNA gene, partial sequence	2713	2713	100%	0.0	99%	EU622630.1

### 3.2. Effect of Time on Biosorption Process

Initial metal ion concentration plays a role in determining the bioaccumulative capacity of the isolated bacterium. As the heavy metal concentrations increased, the cellular growth was inhibited (Figure 2A). Figure 2B illustrates the influence of contact time on the absorption equilibrium of dead and live cells of 139SI at a metal concentration of 50 ppm. In all cases, the absorption capacity was higher in dead cells than in live cells. The uptake rate of non-living biomass significantly increased with increasing contact time and reached 90% of the total biosorption capacity within 50 min (Figure 2B). Subsequently, the *q* value became almost constant. However, the uptake of chromium initially increased and reached 35 mg/g within 50 min, followed by a slower biosorption phase with the live cells. The rate of the biosorption process can be attributed to the presence of various functional groups with different reactions to chromium ions on the cell surface [31]. Hung *et al.* [13] reported that a slow sorption is ascribed to the inner penetration and intercellular accumulation, whereas a fast sorption rate is related to the increase of the binding sites on the cell surface. Based on the results, a contact time of 50 min was set as an equilibrium condition during the experiment. There is no significant change in uptake of chromium by the biosorbent after 50 min, although all the experiments were carried out for up to 350 min. This is implying saturation of the biosorbent at about 50 min.

### 3.3. Effect of pH on Biosorption Process 

**Figure 3:**
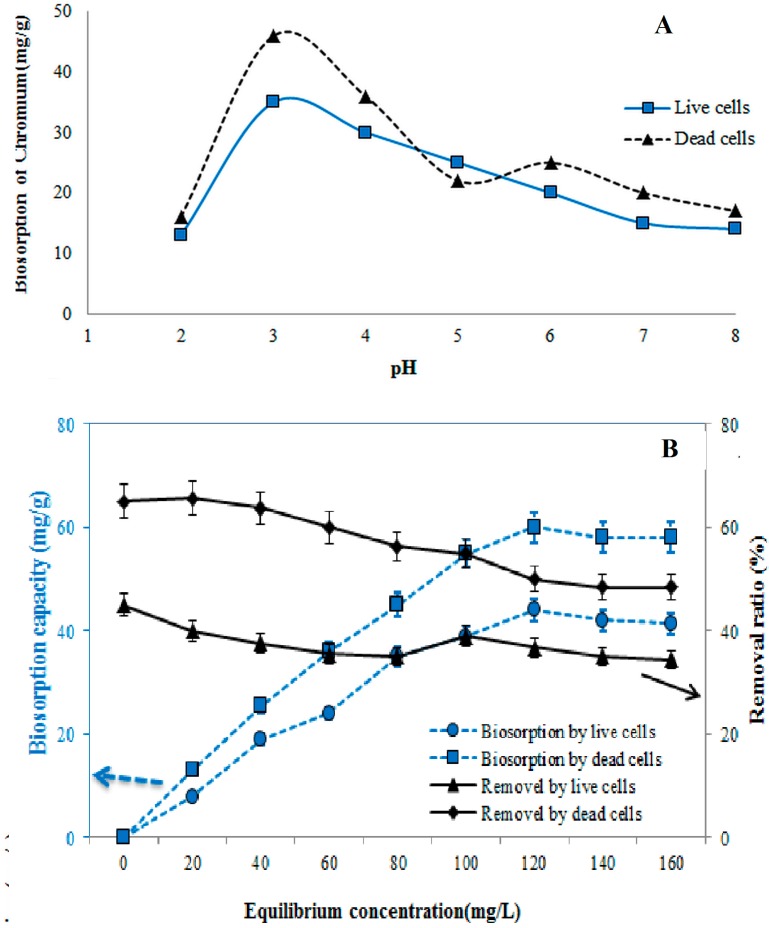
Effect of (**A**) pH and (**B**) initial concentration on Cr(VI) sorption using dead and live cells. Vertical bars indicate SE (*n* = 3).

The biosorption of chromium significantly influenced the pH of the solution for both live and dead cells. The cell surface, degree of ionization, and the chemistry of metal solution were also affected [32,33]. The effect of pH was studied in the range of 2.0 to 8.0 (Figure 3A). The sorption capacity increased from pH 2.0 to 3.0, then drastically decreased with the increasing pH. The maximum rates of biosorption were 46 mg/g of dead cells and 35 mg/g of live cells, respectively, at pH 3.0 (Figure 3A). At pH 7.0 and 8.0, biosorption showed no statistically significant differences. The rates of sorption at pH 3.0 were 92% of dead cells and 70% of live cells, respectively. Since, the pH is related to the net charge on the cell surface, which determines the extent of the cellular sites occupated by protons and other ions of the medium, the competision between heavy metals and H^+^ for same cellular binding sites can result in decrease or increase in cellular heavy metal uptake and toxicity, depending on pH. The chromium removal rate decreased with the increase in pH beyond pH 5, which might be due to the osmotic changes and hydrolyzing effect [34]. Despite the discrepancies recorded in the literature on the effect of pH on the biosorption, it is inferred that pH would alter the process of adsorption of metal ions to cells and it varies with the type of adsorbents (cells) and adsorbates (metal ions) [32,33]. The results of the present study indicated that acidic pH levels favored maximal biosorption of chromium by non-living cells while alkaline condition did not support biosorption. At pH < 3.0, the net charge on the cell wall is positive thereby inhibiting the approach of positively charged ions. Hasan *et al.* [35] reported maximum removal of nickel in the pH range of 4.5–5.5. The variation of adsorption of nickel at various pH is on the basis of metal chemistry in solution and the surface chemistry of the sorbent. The pHmax where maximum removal occurs is related to the pka or the first hydrolysis product of the metal. The decrease in removal of Ni(II) above pH 5 is due to the formation of Ni(OH)_2_. The absorption process in different pH for various heavy metals depends on the interaction of metals with biomass, surface adsorption, chemisorption and complexation, and chemical nature of metals [34]. The decreased sorption rate was also expected at the cell surface due to its negatively charged nature or due to the increase in the ability of protons to compete with chromium ions for the binding sites in the biomass. The rate of chromium uptake and the extent were enhanced as the pH increases up to certain pH range. High alkaline pH value causes reduction in the solubility of metals, which contributes to a low absorption rate. The property of functional groups on the biomass surface is not the only factor that affects the sorption of chromium, but also dependent on the chemical speciation of chromium in the solution [36]. At pH 2.0 to 8.0, the dead cells yielded higher rates of Cr sorption capacity than those exhibited by the live cells because of the large negative surface charge (Figure 3A). At a low pH, the dominant form of Cr(VI) is HCrO^−4^, which can be reduced to Cr(III) in the presence of an adsorbent and attracted through positively charged sites.

### 3.4. Effect of Initial Cr(VI) Concentration on Biosorption Process 

The biosorption experiment was conducted at different initial concentrations (20 mg/L to 160 mg/L) using an optimum contact time of 50 min at pH 3.0 (Figure 3B). The biosorption process in both live and dead cells showed similar trends and increased with the increase of chromium concentration. The results show an increase in the biosorption capacity and hence percent sorption as the initial metal ion concentration increased. The percent sorption was optimized at about 120 mg/L, when equilibrium was attained. This could be attributable to the saturation of the available binding sites on the biosorbent, resulting from increased competition for the fixed number of binding sites on the biosorbent by more and more metal ions, leading to a reduction in the complexation of the metals with the biosorbent. The maximum rate of biosorption depended on the availability of binding sites, which was limited by the increase in initial concentration.

### 3.5. Desorption Efficiency

**Figure 4:**
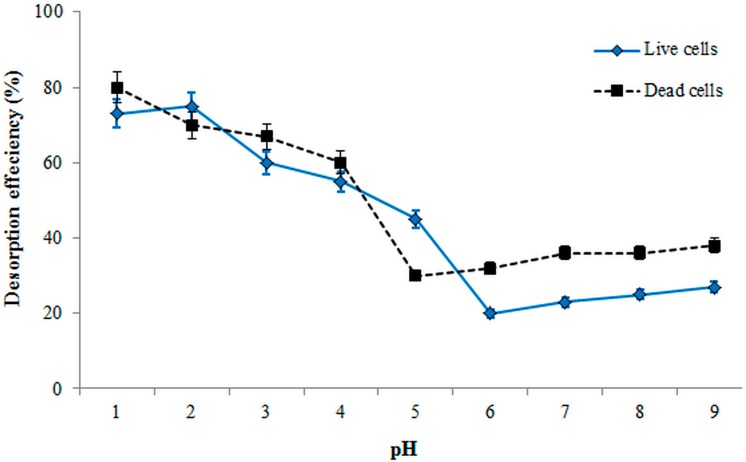
Effect of pH on desorption efficiency.

The potential ability of biomass regeneration and the recovery of metal ions are the most important aspects of the desorption process. Figure 4 illustrates the DE of both live and dead cells in the pH range of 1.0 to 9.0. The DE values of live and dead cells were low after pH 5.0 and 4.0, respectively. The high DE of live biomass demonstrated that more ions were released as the pH decreased from 5.0 and reached 75% at pH 2.0. Statistical analysis indicated that the DE showed no significant difference after pH 6.0 for both cells. However, the DE reached 80% at pH 1.0 in the case of the dead absorbents. The binding strength of the dead cells with ions was also weaker than that of the live cells. Generally, the desorption process varies in different pH and types of biomass.

### 3.6. Biosorption Equilibrium

The Q_max_ values were 12.94 and 20.35 mg/g for live and dead cells, respectively (Table 2). The β constant was also higher in the dead cells (0.728) than in the live cells. In the present work, the dead cells were more efficient, stronger, and better adsorbents than live cells under these experimental conditions. Based on the correlation coefficients (all greater than 0.80), the Langmuir isotherm with high *R*^2^ values presents a more suitable model for both dead and live cells than the Freundlich isotherm. Li *et al.* [27] also reported another equilibrium parameter (R_L_) of Langmuir isotherm, which indicates the biosorption process and the shape of the isotherm (R_L_ = 0: for the irreversible case, R_L_ > 1: for unfavorable case, 0 > R_L_ > 1: for favorable case). R_L_ can be calculated as follows (Equation (8)):

R_L_ = 1/1 + βC _max_ (8)

where C_max_ (mg/L) is the highest chromium concentration. The R_L_ value of live cells (0.257) was higher than that for dead cells (0.015) under the same concentration range, which indicated favorable biosorption of Cr of the dead cells (Table 1).

Freundlich isotherm is based on the *n* and K_f_ values; large amounts of *n* show strong interactions between the metal and the biosorbent [37]. However, the values of *n* and K_f_ in this study were 1.12 and 1.369 for live cells and 2.541 and 4.165 for dead cells (Table 2). The interaction of dead biomass with chrome ions was stronger than that with live biomass, and both types of cells showed favorable chromium uptakes under studied conditions. The *R*^2^ of biosorption (Table 2) for both cells in the Langmuir isotherm was also higher than that for the Freundlich isotherm. Consequently, the live and dead biomasses followed the homogenous surface model through the monolayer biosorption of active surface sites.

**Table 2:** Isotherm parameters for Cr(VI) adsorption onto live and dead cells.

Cells		Langmuir Model			Freundlich Model		
	q_max_(mg/g)	β	R_L_	*R*^2^	n	K_f_ (L/g)	*R*^2^
Live	12.94	0.032	0.257	0.971	1.12	1.369	0.813
Dead	20.35	0.728	0.015	0.966	2.541	4.165	0.933

### 3.7. Kinetic Modeling Studies

**Figure 5:**
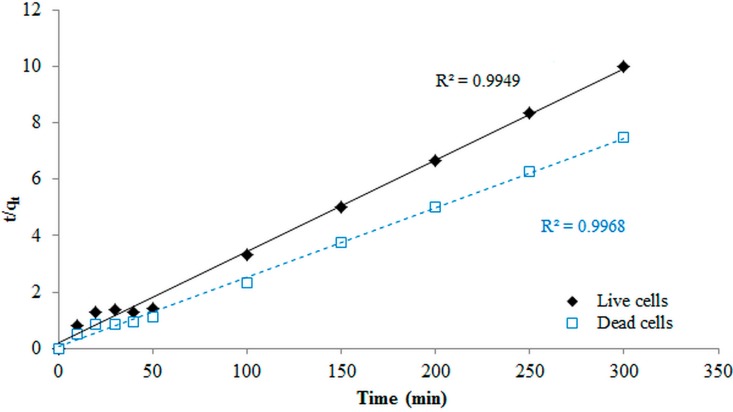
Pseudo-second-order kinetic model for biosorption of Cr(VI).

For the first-pseudo model, the values of Q_e_ and K_1_ were obtained from the slope and intercept of the plot of ln(q_e_–q_t_) *versus t*, whereas the values from the second-pseudo model were calculated from the slope and intercept of a plot t/q_t_ *versus t*. The first-pseudo order showed the lowest coefficients of determination (*R*^2^ = 0.44), which indicates that the actual data for Cr biosorption onto both biomasses did not follow the first-order model. The experimental data for both cells followed the second-order model with coefficient values of 0.994 and 0.996 for live and dead cells, respectively (Figure 5). The rate constants also indicated that the biosorption of Cr by using dead cells was faster than that when using live cells (Figure 5). Moreover, the Q_e_ values were 45.5 and 35 mg/g for the dead and live biomasses, respectively. Therefore, the biosorption of dead and live cells of *B. salmalaya* 139SI in this study followed the second-pseudo-order mechanism. This implies that the biosorption of Cr(VI) onto microbial cell was via chemisorption process and the rate determining step is probably surface biosorption, which explains that the process occurs through valence forces such as sharing or exchange of electrons between adsorbent and Cr(VI) metal ions. This result agreed with that reported by Kumar and Kirthika [38], in which the same conclusion indicated that the second-order equation correlates well with the adsorption studies and corresponds to the chemisorption process. This finding also agreed with the report of Attia *et al.* [28] who reported a good performance of the pseudo-first order in the case of Cr(VI) adsorption from aqueous solutions on treated acid-activated carbon and sawdust, respectively. However, the literature suggests that the kinetic models of adsorption are strongly related to the type of adsorbent material [28,39].

### 3.8. Mechanism Discussion

**Figure 6:**
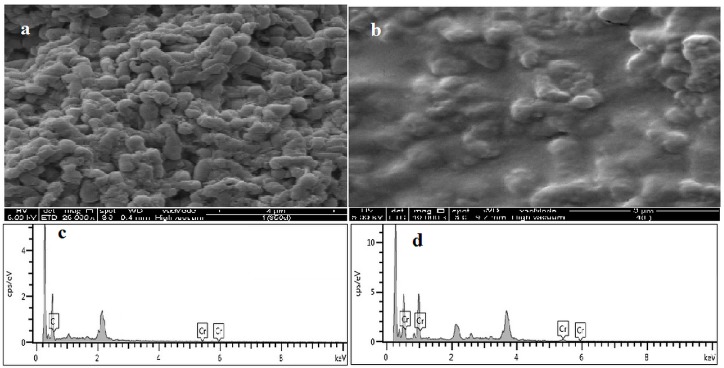
SEM analysis of dead cells (**a**) before adsorption; (**b**) after adsorption; (**c**) EDX analysis of A; and (**d**) EDX analysis of B.

**Figure 7:**
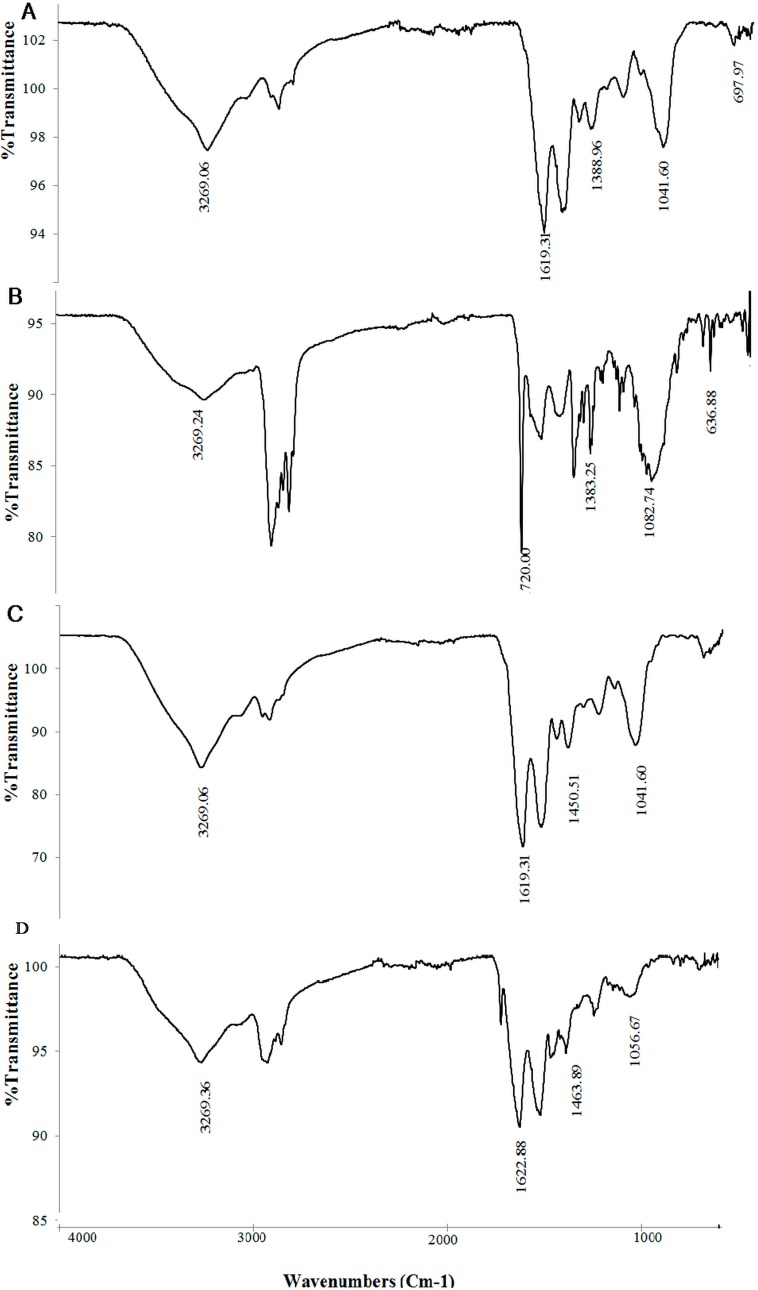
FTIR analysis of dead cells (**A**) before and (**B**) after and live cells and (**C**) before and (**D**) after the chromium adsorption process.

The surface morphology of dead cells analyzed through SEM was illustrated before and after the adsorption process (Figure 6A,B). The morphological aspects of dead biomass before chromium adsorption were long, thin, and rod-shaped. After the absorption of chromium, a cell seriously changes in morphology has been observed (Figure 6B). This phenomenon may be attributed to the coverage of the cell surface with chromium ions, which looked like fat, spongy, and plumped. This finding agrees with the elemental analysis through the EDX spectra of dead cells (Figure 6C,D). EDX is a useful tool for evaluating the elemental and chemical analyses of biosorbents [40]. The percentages of chromium were presented as additional peaks and demonstrated that the chromium ions were attached to the cell surface. This result agrees with the report of Huang *et al.* [13], who observed that Cd(II) accumulated in the surface of dead cells. By contrast, Cd(II) was damaged in the live cell surface, which resulted in the release of cadmium back into the solution. FTIR spectra analyses of live and dead cells were conducted to obtain information regarding the possible functional groups involved in the Cr(VI) adsorbents. The FTIR analysis was measured in the range of 600 cm^−1^ to 4000 cm^−1^ wave numbers for all the adsorbents before and after the adsorption process (Figure 7A–D). A significant shift in the adsorption peaks can be seen by comparing the FTIR spectra of the live and dead cells without chromium ions. Attia *et al.* [28] indicated that the display number of adsorption peak shows the nature of the adsorbents. The strong band around 3269.06 cm^−1^ represents the binding of intermolecular bonded hydroxyl groups (O–H) with chromium ions. The peaks around 2959.17 cm^−1^ indicate the C–H bonds of (–CH_2_) groups. The peak observed around 1619.31 cm^−1^ is attributed to the carbonyl and γ-pyrone groups with strong bonds of C=C and C=O.

At 1041.60 cm^−1^, C–O and –OCH_3_ showed a strong bond (Figure 7). The adsorption peaks around 697.97 cm^−1^ corresponds to the present aromatic compounds. However, the spectral graph demonstrated different characteristics in the peaks after absorption of chromium using the biomass. The C=O bending vibration shifted from 1625.01 cm^−1^ to 1622.88 cm^−1^ in live cells and 1619.31 cm^−1^ to 1622.34 cm^−1^ in dead cells. After the adsorption process, the C–H stretching bonds of dead and live cells shifted to 2945.20 and 2929.27 cm^−1^, respectively. In parallel, 1041.60 cm^−1^ was observed for both live and dead cells before the chromium adsorption in the O–H (hydroxyl) and C–O stretching. By contrast, they changed to 1056.67 cm^−1^ in live cells and 1082.74 cm^−1^ in dead cells after the sorption process. These changes can be attributed to the interaction between organic functional groups and metals on the cell wall [12,41,42].

### 3.9. Bioaccumulation of Chromium by Strain 139SI

**Figure 8:**
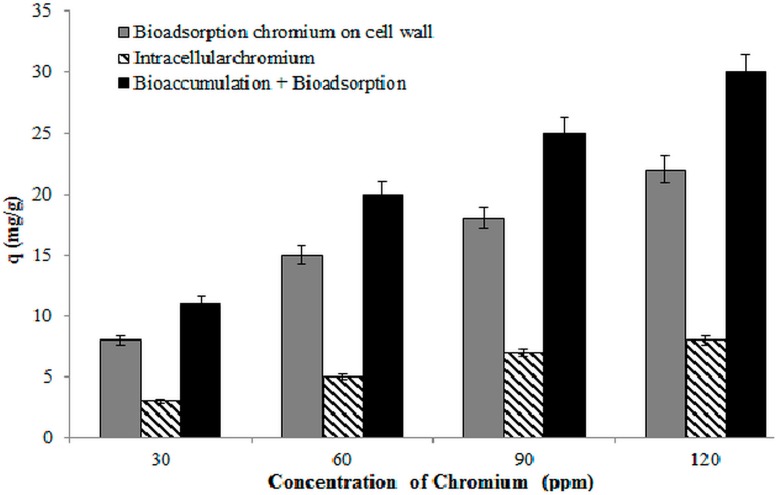
Bioaccumulation of chromium using *B. Salmalaya* 139SI. (pH, 3.0; concentration, 50 mg/L; adsorbent dose, 1 g/L).

Figure 8 shows the result of chromium accumulation using *B. salmalaya* 139SI at different initial concentrations of Cr(VI). The majority of the chromium ions were adsorbed onto the cell wall compared with the bio-accumulated inside the cells. These results are similar to those of some researchers [13,43,44]. Pabst *et al.* [43] recorded the high accumulation of cadmium and lead ions on the cell surface. They reported that surface accumulation works as the protection of the cell by restricting its contact with the outer membrane layer. Our observation indicated that the surface sorption is enhanced as the metal concentration increases, which is possibly related to the cell protective strategy and cell-surface modification. However, our finding showed that chromium accumulation occurred in the cell wall of *B. salmalaya* 139SI rather than intracellular accumulation, which is probably ascribed to the detoxifying mechanism in the *B. salmalaya* 139SI. The genus *Bacillus* showed a significant absorption capacity because of their composition of spores and multiple additional envelopes. The presence of surface structures in the *Bacillus* cell wall, such as paracrystalline, proteins, and exopolysaccharide, supported the interaction with metal ions. The family *Bacillaceae* can also survive in harsh physical and chemical conditions by changing the form of spores. Research has proven the presence of surface-layer proteins and enzyme-binding metals in this structure. Allievi *et al.* [45] indicated the direct correlation between the metal biosorption capacity and the surface layer protein content. 

### 3.10. Biosorption Thermodynamics

**Figure 9:**
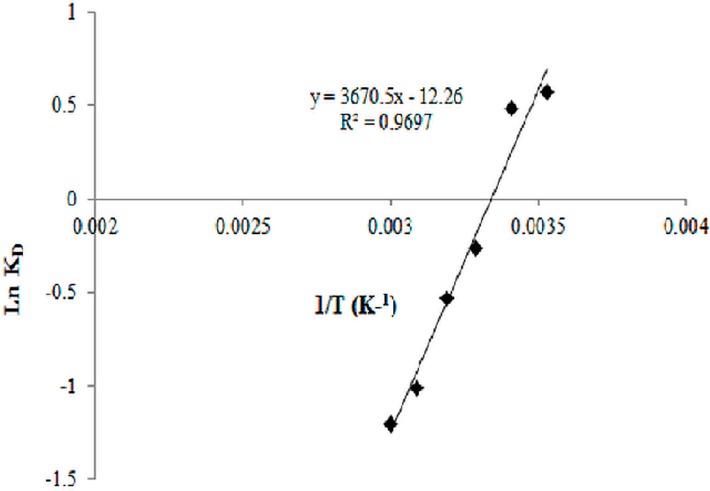
Estimation of thermodynamic parameters for chromium biosorption onto *B. Salmalaya.*

Figure 9 shows the lnK against 1/T plot. The ΔS^0^ and ΔH^0^ parameters can be calculated from the intercept and slope of the plot, respectively. ΔG^0^ (KJ/mol) was calculated as −1.35, −1.17, +0.667, +1.38, +2.72, and +3.34 for the biosorption of chromium at 20 °C, 30 °C, 40 °C, 50 °C, 60 °C, and 80 °C, respectively. The negative values of free energy demonstrated the spontaneous nature of adsorption and the thermodynamic feasibility of the sorption [46,47]. Furthermore, the ΔS^0^ and ΔH^0^ parameters were 0.101 and −30.54 kJ·mol^−1^·K^−1^, respectively. The ΔH^0^ values provide information about the type of biosorption, which is either chemical or physical. If the ΔH^0^ ranging from 2.1 kJ·mol^−1^ to 20.9 kJ·mol^−1^ represents a physical sorption, the values ranging from 20.9 kJ·mol^−1^ to 418.4 kJ·mol^−1^ indicate the biosorption process through chemical sorption [48]. The negative and positive values of enthalpy also reveal the endothermic nature of the adsorption in the exothermic nature of the biosorption processes. However, the positive value of entropy indicates the increased randomness at the solid solution interface during the fixation of the ions onto the active sites of the biosorbent. Thus, our results on thermodynamic evaluation indicate that the mechanism of Cr(VI) adsorption is endothermic; that is, chemisorption.

### 3.11. Comparison Of Dead and Live Biosorbents

Based on the kinetic analysis and the equilibrium, dead cells showed a high rate of biosorption capacity and indicated a more efficient biosorbent compared with live cells. The high DE of dead biomass suggests the potential of dead cells to recover and remove the chromium ions compared with live cells. Similar results on the sorption of heavy metals using non-living and living biomasses of various bacteria have been reported by researchers, such as Velásquez, L. and Dussan [49], who applied *Bacillus sphaericus*; Zhou *et al.* [50] used *Bacillus lichenniformin*; and Sedighi *et al.* [51] applied *Lactobacillius bulgaricus.* FESEM and FTIR analyses were conducted to illustrate the chromium that significantly damaged both cells and suggested that more functional groups were involved in the absorption process. However, further work is necessary for the exegesis of the biosorption phenomenon. Many researchers have reported that dead biomass demonstrated advantages compared with live cells and caused the increase of the cell surface area [13,27]. Moreover, no nutrient supplies are needed when the cell surface is large, and dead cells are more suitable for the removal and biosorption of Cr(VI) from aqueous solution. According to the literature, *Bacillus* genus is classified as a source of metal adsorbent because of the composition of their spores and multiple additional envelopes [45].

## 4. Conclusions

Langmuir isotherm fitted well with the adsorption process with a maximum sorption capacity of 20.35 mg/g. FTIR and SEM analyses indicated that non-living cells are premier in the sorption of Cr(VI) compared with live cells under the experimental conditions. The chromium ions were adsorbed onto the cell wall followed by bio-accumulation inside the cells. The thermodynamic parameters illustrated the spontaneous and endothermic nature of biosorption process. Therefore, the present results illustrated the potential of *Bacillus salmalaya* 139SI for enhancing the sorption capacity with a high recovery of Cr(VI) by using dead biomass within a short period of time. This indicated that strain 139SI could have the possibility of developing a resistance mechanism to deal with chromium toxicity. The bacterial population in the test system could also have developed resistance to several undesirable conditions and maintains physiological traits that could benefit microbial survival and maintenance in contaminated environments. 
